# Diagnostic accuracy of a screening electronic alert tool for severe sepsis and septic shock in the emergency department

**DOI:** 10.1186/s12911-014-0105-7

**Published:** 2014-12-05

**Authors:** Sami Alsolamy, Majid Al Salamah, Majed Al Thagafi, Hasan M Al-Dorzi, Abdellatif M Marini, Nawfal Aljerian, Farhan Al-Enezi, Fatimah Al-Hunaidi, Ahmed M Mahmoud, Ahmed Alamry, Yaseen M Arabi

**Affiliations:** Emergency Medicine and Intensive Care Department, King Saud Bin Abdulaziz University for Health Sciences, Riyadh, Saudi Arabia; Emergency Medicine Department, King Saud Bin Abdulaziz University for Health Sciences, Riyadh, Saudi Arabia; Intensive Care Department, King Saud Bin Abdulaziz University for Health Sciences, Riyadh, Saudi Arabia; Quality Management Department, King Saud Bin Abdulaziz University for Health Sciences, Riyadh, Saudi Arabia; King Abdullah International Medical Research Center, Riyadh, Saudi Arabia; Clinical Information Management Systems, King Saud Bin Abdulaziz University for Health Sciences, Riyadh, Saudi Arabia; College of Public Health and Health Informatics and Quality Management & Consultant of Emergency Medicine, King Saud Bin Abdulaziz University for Health Sciences, Riyadh, Saudi Arabia; Intensive Care Department, College of Medicine, King Saud bin Abdulaziz University for Health Sciences, PO Box 22490, Mail code 1425, Riyadh, 11426 Saudi Arabia

**Keywords:** Clinical decision support, Sepsis, Sensitivity and specificity, Septic shock, Emergency department, Electronic alert

## Abstract

**Background:**

Early recognition of severe sepsis and septic shock is challenging. The aim of this study was to determine the diagnostic accuracy of an electronic alert system in detecting severe sepsis or septic shock among emergency department (ED) patients.

**Methods:**

An electronic sepsis alert system was developed as a part of a quality-improvement project for severe sepsis and septic shock. The system screened all adult ED patients for a combination of systemic inflammatory response syndrome and organ dysfunction criteria (hypotension, hypoxemia or lactic acidosis). This study included all patients older than 14 years who presented to the ED of a tertiary care academic medical center from Oct. 1, 2012 to Jan. 31, 2013. As a comparator, emergency medicine physicians or the critical care physician identified the patients with severe sepsis or septic shock.

In the ED, vital signs were manually entered into the hospital electronic heath record every hour in the critical care area and every two hours in other areas. We also calculated the time from the alert to the intensive care unit (ICU) referral.

**Results:**

Of the 49,838 patients who presented to the ED, 222 (0.4%) were identified to have severe sepsis or septic shock. The electronic sepsis alert had a sensitivity of 93.18% (95% CI, 88.78% - 96.00%), specificity of 98.44 (95% CI, 98.33% – 98.55%), positive predictive value of 20.98% (95% CI, 18.50% – 23.70%) and negative predictive value of 99.97% (95% CI, 99.95% – 99.98%) for severe sepsis and septic shock. The alert preceded ICU referral by a median of 4.02 hours (Q1 - Q3: 1.25–8.55).

**Conclusions:**

Our study shows that electronic sepsis alert tool has high sensitivity and specificity in recognizing severe sepsis and septic shock, which may improve early recognition and management.

## Background

Severe sepsis and septic shock are responsible for significant morbidity and mortality. In the United States, sepsis mortality reportedly occurs in 65.5 per 100,000 persons [1]. In Europe, the in-hospital mortality rate from sepsis is estimated to be 24.1% [2]. Notably, the incidence of sepsis has dramatically increased in recent years, with the sepsis rate per 10,000 admissions doubling between 2000 and 2008 [3]. Moreover, in-hospital deaths are reported to be eight times greater for patients hospitalized for sepsis compared to those hospitalized with other diagnoses (17% and 2%, respectively) [3]. Sepsis-related mortality is also extremely costly to the healthcare system, with an estimated $14.6 billion in 2008 in USA [3]. In Europe, 25% of sepsis patients are admitted to the intensive care unit (ICU) through the emergency department (ED) [2].

Compliance with evidence-based guidelines for severe sepsis and septic shock management has been shown to be low, which has been attributed to factors such as delayed recognition [3]. Thus, a number of international campaigns (e.g., Surviving Sepsis Campaign, World Sepsis Day) have been launched to raise awareness, improve the care of patients with severe sepsis and septic shock, and emphasize early identification and intervention, which have been shown to reduce mortality [4,5]. Furthermore, the 2012 Surviving Sepsis Campaign guidelines recommended routine screening for severe sepsis to allow earlier implementation of therapy and stated that the “key to achieving a reduction in mortality from severe sepsis is not just standardized evidence-based treatment, but equally important, the early recognition of sepsis” [6].

A significant challenge in recognizing severe sepsis early is the complexity of the sepsis presentation, which makes it significantly more challenging to identify patients compared to many other time-critical conditions that are treated in the ED, such as ST-segment elevation myocardial infarction or acute ischemic stroke. Another challenge is ED crowding, which has been linked to the decreased likelihood of adherence to guideline-concordant care. Studies have also demonstrated a relationship between ED crowding and delayed antibiotic administration [7]. A promising measure in improving the efficacy of the health care services provided in ED during crowdedness periods [8]. Such systems should be time efficient and easy to use in order to maintain their impact even during periods of ED crowding.

Different screening tools using different combinations of severe sepsis and septic shock criteria have been studied in ED patients, with sensitivities ranging from 14 to 69% and specificities ranging from 35 to 99% [9-11].

To be effective, a screening tool should meet a number of important criteria [12]. It must cause little or no patient morbidity, be affordable and easily available, identify the conditions for which treatment exists and be more effective when applied early in the disease course [13]. Sepsis is a prime example of a disease in which a screening tool could significantly impact ED management. The objective of this study was to determine the diagnostic accuracy of an electronic alert system in detecting severe sepsis or septic shock in (ED) patients.

## Methods

### Study setting

This single-center study of an electronic sepsis alert tool was conducted at a Joint Commission International (JCI)-accredited tertiary care academic medical center. This initiative was a part of a hospital-wide quality-improvement project that addressed the care of patients with severe sepsis and septic shock. The study was performed over a 4-month period from Oct. 1, 2012 to Jan. 31, 2013. The hospital has 900 beds and receives approximately 200,000 ED visits each year. ED admissions represents 46% of the total hospital admissions per year.

In the ED, vital signs were manually entered into the hospital information system (QuadraMed Computerized Patient Record System, Reston, VA, USA) hourly in the critical care area of the ED (patient triage levels one or two based on the Canadian Triage And Acuity Scale [CTAS]) and every 2 hours in other areas in the ED (patient CTAS triage levels three and four) [4]. The ED had a stat laboratory with a turnaround time for white blood cell and lactate tests of approximately 1 hour. Patients presenting with severe sepsis and septic shock to the ED were first seen by the ED staff and then treated according to their clinical conditions. The patients were then were referred to the ICU team or to other services, as necessary.

### Sepsis screening tool development

To develop a sepsis-screening tool, we first began a multidisciplinary sepsis-working group to develop a severe sepsis and septic shock alert for patients older than 14 years of age. The group used multiple “Plan, Do, Study, Act” (PDSA) cycles to test different combinations and determine appropriate detection parameters [14]. After multiple PDSA cycles in the development (testing) domain of our electronic health record (EHR) system, the combination shown in Sepsis-screening tool alert parameters appeared to have the best ability to screen for severe sepsis and septic shock in an ED setting. This tool works as follows.

**Figure 1:**
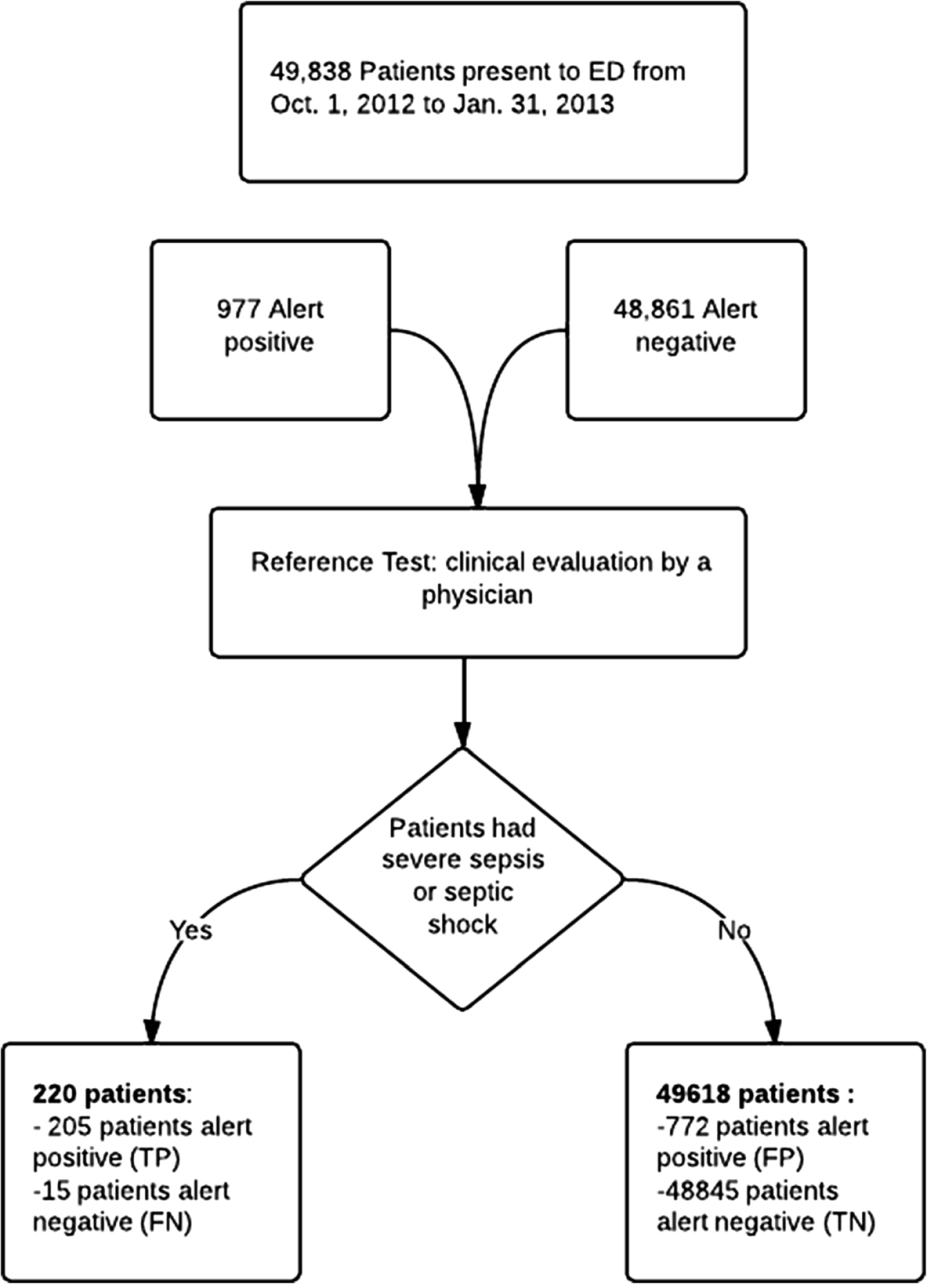
**Patient study inclusion pathway.**

1. The screening tool automatically scans certain clinical and laboratory parameters, as well as the physician orders for fluid bolus or oxygen therapy (List 1).
2. If certain conditions are met (Sepsis-screening tool alert parameters), the system generates a “severe sepsis and septic shock” alert, and the test is considered to be positive.
3. This alert goes to the “nurse work list”.
4. If the criteria are not met, the test is considered to be negative (Figure 1).
5. The nurse responds to the alert and notifies a physician using a paging system, as instructed in the alert message.
6. To avoid multiple activations on the same patient, the alert is deactivated as follows:
  i. for 48 hours if the patient has suspected severe sepsis and septic shock,
  ii. for 24 hours if the patient does not have severe sepsis or septic shock, and
  iii. indefinitely if the code status precludes intensive care management of sepsis.
7. Alerts do not occur during deactivation time.

### List 1: Sepsis-screening tool alert parameters

***Two of the systemic inflammatory response syndrome criteria***

- Temperature >38°C or <36°C
- Pulse >90 beats per minute
- Respiratory rate >20 breaths per minute
- White blood cell count >12,000 or <4,000 ml

**And**

***One organ dysfunction***

- Systolic blood pressure <90 to 86 mm Hg with intravenous fluids or <86 mm Hg regardless of fluids
- Blood oxygen saturation <90% to 85% with supplemental oxygen or <85% without oxygen
- Lactate >2 mmol/L

**OR**

***Two of the above organ dysfunction criteria***

### Study design

The study included a prospective consecutive series of all adult patients presenting to the ED from Oct. 1, 2012 to Jan. 31, 2013. We considered the clinical assessment of the emergency and ICU physicians to be the reference standard. ED and ICU physicians assessed patients for the presence of severe sepsis or septic shock using the standard diagnostic criteria [6]. The assessment was independent from the electronic sepsis alert system. The study excluded patients younger than 14 years of age, which is the cutoff in our hospital for adult patients. The study was approved by the National Guard Health Affairs (NGHA) Institutional Review Board (IRB) and no consent was required.

### Statistical analysis

We calculated the test characteristics of the electronic sepsis alert system, including the sensitivity, specificity, positive predictive value, negative predictive value, positive likelihood ratio, and negative likelihood the ratio with corresponding 95% confidence intervals (CIs). For patients’ who were referred to ICU, we collected additional demographic and clinical data. In addition, we calculated the time from alert activation to the ICU referral and values were expressed as medians and quartiles. We used the SPSS 20.0 software package (SPSS Inc., Chicago, IL, USA) for statistical analysis.

## Results

### Patients

In the 4-month study period, 49,838 patients presented to the ED, of whom 220 were identified by the ED or ICU physicians to have severe sepsis or septic shock. Table 1 shows the true positive and true negative test results. Table 2 shows the demographic and clinical characteristics of patients admitted to ICU.

**Table 1:** **Test results of patients with severe sepsis or septic shock documented by an ED physician, ICU referral, or both**

		**Patients with severe sepsis or septic shock (based on ED physician or ICU team)**		
		**Positive**	**Negative**	**Total**
**Sepsis alert system**	Positive	True positive	False positive	
		205	772	977
	Negative	False negative	True negative	48,861
		15	48,846	
	Total	220	49,618	49,838

**Table 2:** **Demographic and clinical characteristics of the patients who were referred to ICU services**

**Characteristics no. (%)**	**n = 163**
**Classification, no. (%)**	
Severe sepsis	81 (49.7)
Septic shock	82 (50.3)
**Signs and symptoms, no. (%)**	
Hyperthermia >38 C (101.0 F)	41 (25.2)
Hypothermia <36 C (96.8 F)	4 (2.5)
Acutely altered mental status	34 (20.9)
Chills and rigors	6 (3.7)
Tachycardia (> 90 bpm)	141 (86.5)
Tachypnea (> 20 bpm)	145 (89.0)
Hypotension (SBP <90 mm Hg or MAP <65 mm Hg)	114 (69.9)
Hypoxia (< 90%)	66 (40.5)
**Laboratory findings, no. (%)**	
Leukocytosis (WBC count >12.000 uL-1)	73 (44.8)
Leukopenia (WBC count <4000 uL-1)	4 (2.5)
Increased creatinine >2.0 mg/dL (176.8 mmol/L) or urine output < 0.50 ml/kg/hour for 2 hours	4 (2.5)
Thrombocytopenia (platelet <100,000)	0 (0.0)
Hyperbilirubinemia (bilirubin > 2mg/dL (34.2 mmol/L))	1 (0.6)
Hyperlactatemia (Lactate > 2 mmol/ L (18.0 mg/dL))	44 (27.0)
Coagulopathy (INR>1.5 or > 60 sec)	2 (1.2)
**Source of sepsis, no. (%)**	
Pneumonia	77 (47.2)
Urinary tract infection	20 (12.3)
Acute abdominal infection	7 (4.3)
Soft tissue infection	4 (2.5)
Other infections	45 (27.6)
**Mechanically ventilated, no. (%)**	43 (26.4)
**Vasopressors, no. (%)**	78 (96.3)
**Lactate, mmol/L mean ± SD**	3.4 ± 2.4
**Glucose, mmol/L mean ± SD**	10.3 ± 4.5
**ICU LOS (day), mean ± SD**	8.3 ± 7.9

### Test characteristics

Table 3 shows the test characteristics of the electronic sepsis alert. For recognizing severe sepsis and septic shock, the test had a sensitivity of 93% (95% CI = 89%–96%), specificity of 98% (95% CI = 98.3%–98.5%), positive predictive value of 20% (95% CI = 18%–23%), and negative predictive value of 99.9% (95% CI = 99.95%–99.98%). The positive likelihood ratio was 59.88 (95% CI, 55.36–64.78), and the negative likelihood ratio was 0.069 (95% CI, 0.429–0.11). The electronic sepsis alert preceded ICU referral by a median of 4.02 hours (Q1–Q3, 1.25–8.55 hours).

**Table 3:** **Test characteristics of the electronic sepsis alert system**

**Property**	**Value**
Sensitivity	0.93 (95% CI, 0.89–0.96)
Specificity	0.98 (95% CI, 0.98–0.98)
Positive predictive value	0.21 (95% CI, 0.18–0.23)
Negative predictive value	0.99 (95% CI, 0.99–0.99)
Positive likelihood ratio	59.88 (95% CI, 55.36–64.78)
Negative likelihood ratio	0.07 (95% CI, 0.04–0.11)

## Discussion

In this study, we described the test characteristics of an electronic screening tool for severe sepsis and septic shock in the ED. The electronic sepsis alert had high sensitivity and specificity and negative predictive value. The low positive predictive value probably resulted from the detection criteria used in the alert system, which did not include clinical information, such as presenting symptoms or confirmatory laboratory tests. In addition, we demonstrated that our electronic sepsis alert tool preceded ICU referral for severe sepsis or septic shock. This finding is important and would facilitate time-sensitive sepsis management and early sepsis care beginning in the ED.

Various tools to screen for sepsis and severe sepsis have been previously evaluated in the ED setting. In a prospective observational study by Meurer et al., an electronic alert was sent to the care team if two or more systemic inflammatory response syndrome (SIRS) criteria were detected in patients older than 70 years. Their system had a sensitivity of 14% and a specificity of 98% for detecting an infection [9]. Nelson et al. used an automated messaging system that alerted the care team if a patient presented to the ED with two or more SIRS criteria in addition to two systolic blood pressure readings of <90 mm Hg. Their system had a sensitivity of 64%, specificity of 99%, positive predictive value of 54%, and negative predictive value of 99% [10]. Jaimes et al. also found that for patients presenting to the ED, the presence of two or more SIRS criteria had a sensitivity to detect infection of 69%, specificity of 35%, positive predictive value of 90%, and negative predictive value of 12% [11].

The main difference between our study and previous studies is the criteria for activating the alert system. We used a combination of clinical and laboratory parameters and a physician order of fluid or oxygen therapy. We used an electronic alert rather than a paper-based system and applied this system in an ED setting. Our results demonstrated that the electronic alert preceded ICU referral by a median of 4.02 hours (Q1–Q3, 1.25–8.55 hours). The low prevalence found in our study was because the alert system screened all patients who presented to the ED, not only the high-risk patients or those who presented to the ED with infection. Alert systems should focus on preventing active failures and individual error-producing conditions [15]. Therefore, achieving a high specificity of 98.4% was important. To avoid “alert fatigue and overriding”, the alert should be followed by directing the bedside nurse to ask the physician to respond to it [16].

Our results must be interpreted based on their strengths and limitations. We demonstrated good diagnostic properties in an ED with a high number of visits. One of the limitations of this study is that it was conducted at a single academic medical center. EHR systems vary widely among institutions, therefore it is not possible to comment specifically on the ease with which our sepsis recognition strategy might be used in other institutions. Another limitation is that we described only the characteristics of patients who were admitted to the ICU. Another factor is related to the limitation of the alert design: our alert system scans the most recent vital signs for sepsis criteria; hence, the trigger threshold is decreased because all criteria must be aligned at the same time.

## Conclusion

The high specificity and sensitivity and negative predictive values of the alert system are promising. We have demonstrated a feasible approach that allows us to recognize patients with severe sepsis with high sensitivity and specificity. An electronic alert preceding ICU referral could lead to earlier sepsis management and minimize delays in recognizing sepsis.

## Abbreviations

ICU: Intensive care unit
ED: Emergency department
CTAS: Canadian Triage and Acuity Scale
PDSA: Plan, Do, Study, Act
IRB: Institutional review board
NGHA: National Guard Health Affairs
CIs: Confidence intervals
SIRS: Systemic inflammatory response syndrome
EHR: Electronic health records
